# Immunoglobulin Y protects intestinal epithelium and modulates gut microbiota

**DOI:** 10.20517/mrr.2025.104

**Published:** 2026-04-21

**Authors:** Shahna Fathima, Tina Sarkar, Navneet Sharma, Paul E Kilgore, Huan H Nguyen

**Affiliations:** ^1^IGY Immune Technologies & Life Sciences, Airdrie T4A 2G8, Canada.; ^2^Eugene Applebaum College of Pharmacy and Health Sciences, Wayne State University, Detroit, MI 48201, USA.; ^3^Department of Dermatology, University Medical Center of the Johannes Gutenberg University, Mainz 55131, Germany.

**Keywords:** IgY, gut microbiota, adhesion assay, cytokines, barrier integrity, passive immunotherapy, adherent pathogenic *E. coli*

## Abstract

**Aim:** This study investigated the effects of Muno-IgY®, a multi-pathogen-specific immunoglobulin Y (IgY), on microbial growth, adhesion, fermentation activity, and immune signaling using a multi-tiered *in vitro* approach.

**Methods:** IgY activity was first evaluated in Caco-2 adhesion and invasion assays using adherent-invasive *Escherichia coli* (AIEC) at optimized concentrations, followed by assessment in a SHIME® *in vitro* gut model inoculated with human fecal microbiota enriched in Enterobacteriaceae. Microbial composition, fermentation markers, and metabolite production were analyzed, and downstream effects on epithelial barrier integrity and immune signaling were evaluated using a Caco-2/peripheral blood mononuclear cell (PBMC) co-culture model exposed to SHIME® effluents.

**Results:** Muno-IgY® significantly reduced AIEC adhesion/invasion from 39.76% in controls to 13.08% and 9.13% at 3 and 6 mg/mL, respectively (*P* < 0.05). In the SHIME® model, IgY significantly increased acetate and propionate production (*P* < 0.05), alongside a marked increase in ammonium concentration (*P* < 0.01). Microbial biomass increased modestly, while alpha- and beta-diversity indices were not significantly altered. The compositional shifts indicated enrichment of beneficial and mucin-associated taxa and reduction of opportunistic or pathogenic species in the Muno-IgY® group. In Caco-2/PBMC co-cultures, IgY-treated effluent decreased transepithelial electrical resistance (TEER) indicating reduced barrier integrity (*P* < 0.05) but significantly decreased pro-inflammatory cytokines Interferon-γ (IFN-γ) and Interleukin-22 (IL-22) (*P* < 0.05).

**Conclusions:** Muno-IgY® demonstrates the ability to inhibit pathogen adhesion and modulate microbial composition and immune responses *in vitro*. These findings support its potential as a non-antibiotic approach for microbiome-targeted interventions, although further validation *in vivo* is required.

## INTRODUCTION

The human gut microbiome plays a crucial role in maintaining gastrointestinal health by supporting normal physiological functions, including digestion, nutrient absorption, energy production, and vitamin synthesis. Beyond these functions, the gut microbiome also contributes to immune modulation by regulating inflammatory responses, producing bioactive metabolites such as short-chain fatty acids (SCFAs), which have anti-inflammatory properties and support epithelial barrier integrity by serving as an energy source for enterocytes, and competing with pathogenic microbes for attachment sites and nutrients, thereby preventing pathogen colonization and subsequent enteric infections^[1,2]^.

Disruptions to this complex ecosystem have been implicated in a range of structural and functional disorders, including gastroesophageal reflux disease (GERD), irritable bowel syndrome (IBS), inflammatory bowel diseases (IBD), and autoimmune coeliac disease^[3]^. Although different patterns of microbial colonization are associated with different disease states compared to healthy controls, a causal relationship between the changes in specific microbes and disease states has not been established^[4]^. Despite this uncertainty, considerable research has focused on microbiome-targeted interventions, including the use of probiotics, prebiotics, and passive immunotherapies to restore the microbial balance of the gut during disease conditions.

Adherent-Invasive *Escherichia coli* (AIEC) represents a distinct group of *Escherichia coli *(*E. coli*) pathobionts that have been functionally associated with inflammatory bowel disease, particularly Crohn’s disease^[5]^. Unlike commensal *E. coli*, AIEC strains are defined by their ability to both adhere to and invade intestinal epithelial cells and to survive and replicate within macrophages, leading to sustained inflammatory responses and disruption of barrier integrity. Interaction of AIEC with epithelial cells triggers production of pro-inflammatory cytokines, enhances epithelial permeability, and contributes to chronic mucosal inflammation^[5]^. Because these behaviors are central to AIEC pathogenesis and disease progression, inhibition of AIEC adhesion and invasion represents an important therapeutic target when evaluating candidate interventions aimed at modulating host-microbe interactions.

Immunoglobulin Y (IgY) is the avian counterpart of mammalian IgG and is a polyclonal immunoglobulin harvested non-invasively from egg yolks. IgY has gained attention as an oral passive immunotherapy^[6]^ due to its safety and feasibility in production. IgY has been used for the passive immunization and treatment of enteric infections such as *Helicobacter pylori* gastritis in humans^[7]^, human norovirus infection^[8]^, *Candida albicans* in mice^[9]^, and parvovirus infection in dogs^[10]^. Its ability to neutralize pathogens and their toxins without disturbing the host microbiota or contributing to antimicrobial resistance makes it a promising tool for gut-targeted immunotherapy^[6]^.

Although studies have demonstrated IgY’s efficacy in inhibiting bacterial adhesion and neutralizing toxins *in vitro* and *in vivo*, its impact on gut microbiota composition, fermentation activity, and host immune responses remains largely unexplored. Moreover, most studies focused on single pathogen-specific IgY. The potential of IgY directed against multiple pathogens and virulence factors, including lipopolysaccharide (LPS) and flagellin, has yet to be fully investigated.

To understand the behavior of IgY within the complex gastrointestinal ecosystem, *in vivo* studies are invaluable. However, such studies are often limited by ethical constraints, high costs, and logistical challenges. As a robust alternative, *in vitro* dynamic fermentation models have been developed to simulate the human gut environment. These models are typically inoculated with fecal microbiota from human donors selected for specific microbial profiles, allowing for controlled and reproducible experimentation. One such well-established and widely used system is the Simulator of the Human Intestinal Microbial Ecosystem (SHIME®), which enables detailed investigation of microbial activity and composition under physiologically relevant conditions^[11,12]^ and has been employed previously to study microbial community composition during nutritional interventions^[12,13]^.

Despite growing interest in IgY as a passive immunotherapeutic, little is known about how IgY influences the gut microbial ecosystem as a whole, including fermentation activity, community structure, and downstream host immune responses. In particular, the effects of multi-pathogen-specific IgY on microbiota-gut-immune crosstalk remain largely unexplored. Here, we address this gap by combining epithelial infection models, a dynamic human gut fermentation system, and immune co-culture assays to provide an integrated assessment of IgY function across multiple biological scales. This approach enables simultaneous evaluation of direct pathogen inhibition and indirect microbiome-mediated host responses, advancing understanding of IgY beyond its established role as a pathogen-neutralizing agent.

In this study, we investigated the effects of Muno-IgY®, a formulation containing multi-pathogen-specific IgY, on gut microbial activity, community structure, and host immune responses using a complementary *in vitro* experimental framework. Specifically, we assessed the ability of Muno-IgY® to inhibit bacterial growth, interfere with the adhesion and invasion of AIEC to intestinal epithelial cells, and modulate gut microbial composition and fermentation activity using a dynamic SHIME® model. In addition, SHIME® effluents were applied to a Caco-2/peripheral blood mononuclear cell (PBMC) co-culture system to evaluate downstream effects on epithelial barrier integrity and cytokine signaling. Together, these integrated approaches provide a comprehensive evaluation of Muno-IgY®’s antimicrobial, microbiota-modulating, and immunomodulatory potential, offering new insights into its role in promoting gut health.

## METHODS

The concentrations of Muno-IgY used in this study were determined based on preliminary in-house screening experiments. Serial dilutions starting at 12 mg/mL were evaluated for *E. coli* growth inhibition on Luria-Bertani (LB) agar plates, from which 3 mg/mL was identified as an effective concentration [Supplementary Data]. Accordingly, a range of concentrations (1.5, 3, and 6 mg/mL), representing half, the optimal, and double the optimal dose, was tested in the AIEC adhesion and invasion assays to assess potential dose-dependent effects. As no significant differences were observed between 3 and 6 mg/mL in these assays, the lower concentration (3 mg/mL) was selected for the SHIME experiment.

Certain experimental procedures were conducted by an external contract research organization (ProDigest, Ghent, Belgium, https://prodigest.eu/technology/shime/), and detailed manufacturer information for all reagents and instruments was not available. Wherever possible, reagent identities and methodological parameters have been specified to ensure reproducibility.

### *In vitro* adhesion assay

The human colon adenocarcinoma cell line Caco-2 (HTB-37; American Type Culture Collection, ATCC) was seeded in 24-wells, coated with 0.1% gelatin, and cultured for 14 days, with three medium changes per week, until a functional cell monolayer with a transepithelial electrical resistance (TEER) was obtained. The cells were maintained in Dulbecco’s Modified Eagle Medium (DMEM) containing glucose and glutamine (Corning®, CA45000-368) and supplemented with N-2-hydroxyethylpiperazine-N-2-ethane sulfonic acid (Sigma-Aldrich, H0887) (HEPES) and 20% (v/v) heat-inactivated fetal bovine serum (FBS).

Adherent-invasive *E. coli* was cultured for 16 h at 37 °C in nutrient broth under aerobic conditions. After incubation, bacteria were quantified by flow cytometry, centrifuged, and resuspended in culture medium containing 1% FBS. Then, AIEC was incubated with the IgY product at 3 concentrations of 1.5, 3, and 6 mg/mL or with the corresponding concentrations of phosphate buffer as a control for 30 min prior to addition to the Caco-2 cells. A 30-min pre-incubation of AIEC with IgY was employed to allow antibody binding to bacterial surface antigens prior to epithelial contact, thereby isolating IgY effects on adhesion and invasion rather than post-attachment killing. Each treatment had three technical replicates (3 wells per treatment). Caco-2 cells were incubated with AIEC incubated with respective doses of Muno-IgY® or PBS for 3 h, after which AIEC were quantified in the culture medium, and the adhered/invaded fraction was estimated from the lysed cells. To assess the invaded fraction, cells were treated with gentamicin before lysis to kill the adhered bacterial fraction^[14]^. The differences in AIEC concentration between the treatments, adhesion/invasion percentage, and invaded fraction were assessed.

### SHIME^®^ reactor study

The SHIME® system (https://prodigest.eu/technology/shime/) was used to evaluate the effects of repeated oral administration of IgY on microbial composition and fermentation markers. The study employed a dynamic *in vitro* fermentation model inoculated with human fecal microbiota to simulate the colonic environment.

#### Donor selection and inoculation

Fecal samples from 20 healthy adult donors were screened via qPCR for Enterobacteriaceae. Inclusion criteria included no known history of chronic gut inflammation and no antibiotic intake during the three months prior to providing the fecal sample. A donor with a high abundance of the taxa was selected. Fecal material collected from the volunteer was cryopreserved at -80 °C following homogenization with anaerobic phosphate-buffered saline and addition of a cryoprotectant solution^[15]^. On the day of inoculation, the frozen sample was thawed and used to prepare a fecal slurry under anaerobic conditions.

A single donor with elevated Enterobacteriaceae abundance was intentionally selected to create a microbiota context enriched in potential IgY target organisms, thereby maximizing sensitivity for detecting IgY-mediated microbial shifts in this proof-of-concept study. While inter-individual variability is a recognized feature of the human microbiome, the use of a single donor enabled controlled, tractable evaluation of IgY effects while minimizing confounding donor-specific variability. Findings should therefore be interpreted as exploratory and donor-dependent.

The collection and use of human-derived samples for this study were conducted in accordance with the Declaration of Helsinki and the Belgian Law of 7 May 2004 concerning experiments on the human person. The study protocol was approved by the Ethics Committee of the University Hospital of Ghent (ONZ-2022-0267) on 12 July 2022. The study involved the voluntary participation of healthy individuals. Participants signed an informed consent.

#### SHIME® reactor setup and conditions

The screening SHIME® consisted of parallel control and treatment arms simulating a single colonic region, each comprising luminal and mucosal compartments. The system was maintained at 37 °C using a water-jacketed setup and flushed daily with nitrogen to maintain anaerobiosis. Reactors were continuously stirred at 300 rpm. The pH was automatically controlled between 6.15 and 6.40. Retention time was set at 32 h, and working volume per colonic reactor was 400 mL.

#### Experimental timeline and treatment

The experiment was conducted over 12 days, beginning with a 3-day stabilization period followed by an 8-day treatment period. An 8-day treatment period was chosen to allow sufficient microbial adaptation to repeated IgY exposure while maintaining system stability and avoiding long-term drift commonly observed in extended *in vitro* fermentations. During stabilization, reactors were fed three times daily with sterile, autoclaved SHIME® feed medium (70 mL per feeding, pH 2.0) and pancreatic juice (30 mL per feeding). During the treatment phase, the test arm received 3 mg/mL IgY with each feeding via the gastric compartment, while the control arm received feed supplemented with control matrix only. The feeding schedule occurred at 9:00, 17:00, and 01:00, with colonic entry approximately 3 h later.

#### Sample collection and analyses

Overall fermentation activity and SCFA production

Luminal samples were collected on days 1, 3, 5, and 8 during the treatment phase for analysis of microbial activity and fermentation markers. The overall fermentative activity was measured in terms of acid/base consumption as an indirect indicator of microbial metabolic activity within the SHIME® system. The consumption of acid (0.5M HCl) and base (0.5M NaOH) was continuously monitored and reported for each colon vessel. The reported parameter shows the acidification of the colonic vessels as calculated by subtracting the acid from base consumption at each time point, indicating overall microbial activity^[16]^. Concentrations of the SCFAs, acetate, propionate, and butyrate were quantified using gas chromatography after liquid-liquid extraction with acetonitrile, with a linear dynamic range of 0.05-50 mM and a limit of detection of 0.02 mM. Lactate concentration was measured using Enzytec**^TM^** D-lactate/L-lactate kit (R-Biopharm)^[17]^ on days 1, 3, 5 and 8 in triplicate from each colon vessel.

Ammonium concentration

Ammonium concentration in the reactor was determined via colorimetric analysis using the indophenol blue spectrophotometric method^[18]^. Each measurement was performed in a single repetition. Samples were collected on days 1, 3, 5, and 8 from each colon vessel from the treatment period onwards.

Microbial community composition

Luminal and mucosal samples were collected on day 8 to assess the microbial composition. Shotgun metagenomic data were processed following the pipeline described previously^[19]^, including quality filtering, trimming, and host genome decontamination with Kneaddata v0.10.0, followed by taxonomic classification using Kraken2 v2.1.3 and Bracken v2.9 against the Genome Taxonomy Database (GTDB). Total bacterial cell counts were determined as described previously^[19]^.

### Caco-2/PBMC co-culture with SHIME® effluent

To evaluate the downstream effects of IgY-modulated microbiota on intestinal epithelial barrier function and immune signaling, samples were collected from the SHIME® reactor on day 8 and applied to an *in vitro* Caco-2/PBMC co-culture system. Day-8 SHIME® effluents were selected to represent microbiota states following sustained IgY exposure, thereby reflecting downstream host responses to stabilized microbial modulation rather than transient effects. This model was used to assess protective effects against inflammation-induced epithelial barrier disruption, measured via transepithelial electrical resistance (TEER), as well as modulation of adaptive immune markers, including Interleukin-17A (IL-17A), Interferon-γ (IFN-γ), Interleukin-21 (IL-21), Interleukin-22 (IL-22), Interleukin-4 (IL-4), and Interleukin-9 (IL-9).

#### PBMC isolation and preparation

PBMCs were isolated from human buffy coats obtained from the Red Cross using Lymphoprep**^TM^** (Axis-Shield). PBMCs were separated by density-gradient centrifugation to remove polymorphonuclear cells (PMNs) and red blood cells. Donors with elevated baseline Interleukin-8 (IL-8) levels were excluded to avoid confounding effects from pre-activated immune cells.

#### Caco-2 cell culture and co-culture setup

Caco-2 cells (HTB-37; ATCC) were seeded onto 24-well semi-permeable inserts and maintained in DMEM supplemented with glucose, glutamine, HEPES, and 20% heat-inactivated FBS. Monolayers were cultured for 14 days, with medium changes three times per week, until a stable barrier with measurable TEER was achieved.

Before co-culture, baseline TEER measurements were recorded (0 h time point). The TEER value of an empty insert was subtracted to correct for background resistance. The inserts bearing Caco-2 monolayers were then placed above PBMCs in the basolateral compartment, which were stimulated with pokeweed mitogen (PWM) to induce an inflammatory response. In the apical chamber, Caco-2 cells were treated with filter-sterilized colonic SHIME® suspensions from either the control or IgY-treated reactors, and simultaneously, the basolateral side was stimulated with PWM for 48 h. Sodium butyrate (Sigma-Aldrich) was included as a positive control for barrier protection, while untreated Caco-2 cells in complete medium served as the negative control. The co-cultures were incubated for 48 h at 37 °C in a humidified atmosphere of 5% CO_2_.

All final TEER values were normalized to their corresponding baseline (0 h) values and were expressed as a percentage of initial resistance to account for inter-insert variability. Basolateral supernatants were collected and analyzed for cytokine levels as described previously^[19]^.

### Statistical analysis

The log-transformed differences in the AIEC concentration, percentage invasion, and the adhesion/invasion percentage between the control and treatments were assessed using one-way ANOVA, and the means were separated using Dunnett’s multiple comparison test. Differences in AIEC concentrations between the IgY product and control groups were assessed using a two-way analysis of nariance (ANOVA) with Tukey’s Honestly Significant Difference (HSD) multiple comparisons test. All statistics were performed using GraphPad Prism version 10.4.0 for Windows (GraphPad Software, San Diego, CA, USA) and R Studio 4.5.1 (R Foundation for Statistical Computing, Vienna, Austria). The adhesion/invasion percentvge and percentage invasion were calculated as follows: 

Adhesion/invasion percentage = adhered/invaded bacteria/(adhered/invaded bacteria + bacteria in the medium) in colony-forming unit (CFU)/mL × 100 

Percentage invasion = invaded bacteria/ (average of adhered/invaded bacteria + average bacteria in medium) in CFU/mL × 100

Differential abundance analysis of microbial taxa was performed using LEfSe and TreeclimbR. For Linear discriminant analysis Effect Size (LEfSe), features with a *P*-value ≤ 0.05 (Kruskal-Wallis and Wilcoxon tests) were considered significant, and Linear Discriminant Analysis (LDA) scores ≥ 2.0 were interpreted as biologically relevant. Taxa with a log_2_ fold change > ± 2 and -log_10_(*P*-value) > 1.3 were classified as both biologically significant. 

To evaluate differences in TEER and immune markers between control and treatment samples, a paired, two-tailed *t*-test was performed on the average of these normalized values, taking the PBMC donors as replicates (number of pairs = 3).

## RESULTS

### *In vitro* adhesion assay

**Figure 1:**
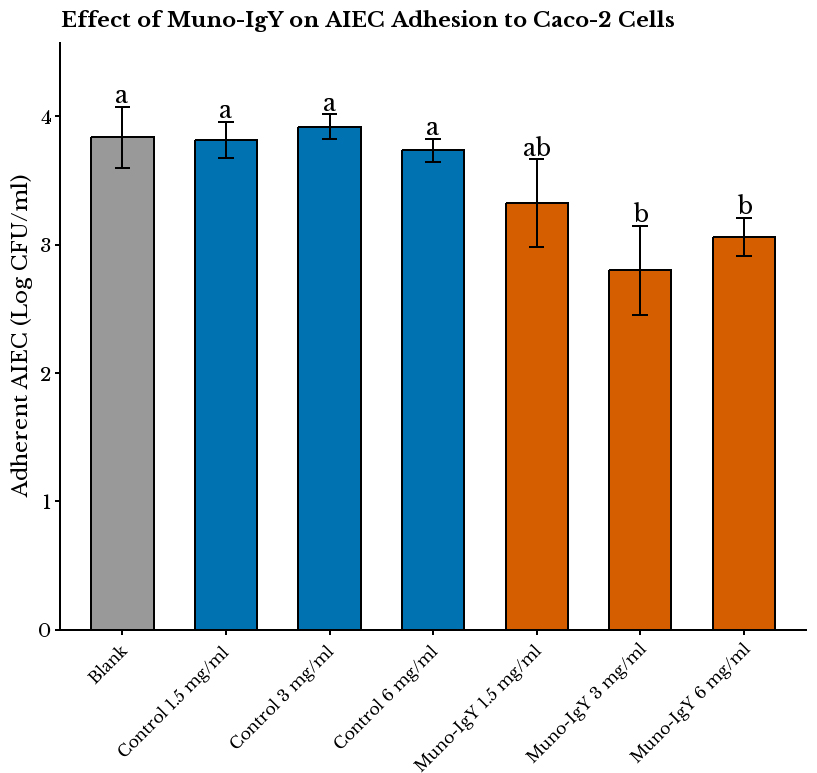
AIEC to Caco-2 cells measured under different treatment conditions. Values are expressed as mean ± SD of log_10_ CFU/mL (*n* = 3 per condition). One-way ANOVA revealed a significant effect of treatment on bacterial adhesion (*P* = 1.02 × 10^-4^). Muno-IgY® at 6 mg/mL and 3 mg/mL significantly reduced AIEC adhesion compared with Blank (*P* = 0.011 and *P* = 0.0009, respectively). Different letters above bars indicate statistically significant differences (*P* < 0.05). AIEC: Adhesion of Adherent-invasive *Escherichia coli*; SD: Standard deviation; CFU: colony-forming unit.

Treatment with Muno-IgY® significantly reduced the number of bacteria recovered from the Caco-2 monolayers and lysates in a dose-dependent manner. Compared to the control, 3 and 6 mg/mL of Muno-IgY® showed a significant reduction (*P* < 0.05) in the adhesion/invasion of AIEC. In the adhesion/invasion fraction, bacterial counts decreased from a control level of 7.79 (± 0.04) log_10_ CFU/mL to 6.94 (± 0.07) log_10_ CFU/mL with 3 mg/mL Muno-IgY® and to 6.60 (± 0.10) log_10_ CFU/mL with 6 mg/mL Muno-IgY®. Similarly, in the invasion fraction, bacterial counts were reduced from 3.83 (± 0.24) log_10_ CFU/mL in the control to 2.80 (± 0.35) log_10_ CFU/mL with 3 mg/mL Muno-IgY® and 3.06 (± 0.15) log_10_ CFU/mL with 6 mg/mL Muno-IgY®. In addition, the concentration of AIEC in the medium was significantly decreased following Muno-IgY® pre-treatment, with the highest inhibition observed at 6 mg/mL [7.61 (± 0.05) log_10_ CFU/mL] compared to the control [7.97 (± 0.09) log_10_ CFU/mL] [Figure 1]. However, 1.5 mg/mL IgY did not have a significant effect on the AIEC concentration in the media. In addition, treatment with 3 mg/mL and 6 mg/mL IgY significantly decreased the percentage adhesion/invasion to 13.08 ± 1.34% and 9.13 ± 3.06%, respectively, compared to the control (39.76 ± 2.51%). Furthermore, there was no statistically significant difference between 3 mg/mL and 6 mg/mL doses, indicating that 3 mg/mL was sufficient to exert a maximal protective effect. Hence, this concentration was selected for subsequent SHIME® experiments.

### SHIME^®^ reactor study

#### Overall fermentation activity

**Figure 2:**
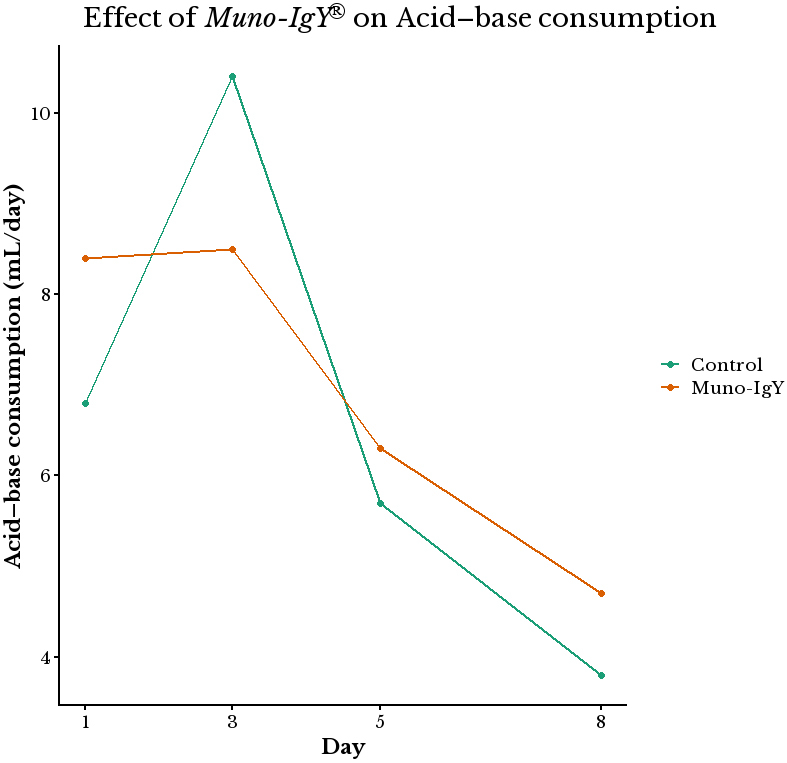
Effect of Muno-IgY® treatment on net acid–base consumption. Muno-IgY® supplementation did not have a significant effect on the acid-base consumption during the study duration (*P* > 0.05) (*n* = 1). IgY: Immunoglobulin Y.

**Figure 3:**
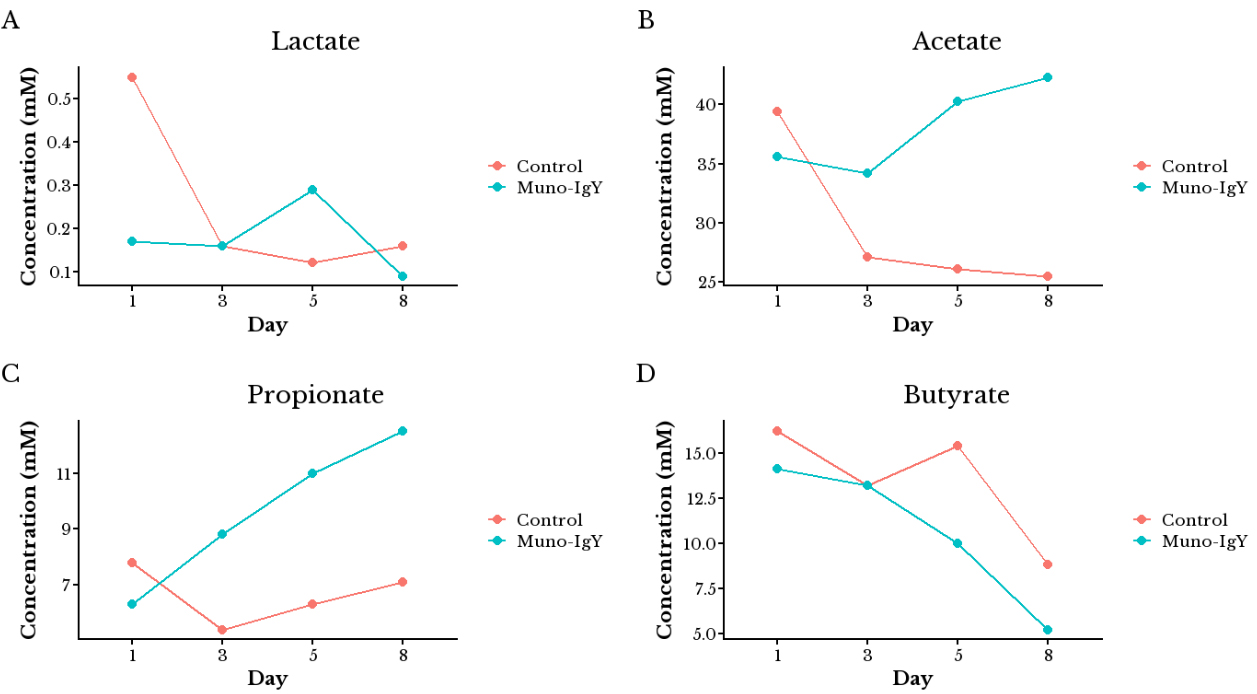
Effect of Muno-IgY® treatment on microbial fermentation activity in the SHIME® model. (A) Lactate concentration; (B) Acetate concentration; (C) Propionate concentration; (D) Butyrate concentration (*n* = 1). IgY: Immunoglobulin Y.

There was no significant effect of the Muno-IgY® treatment on the acid/base consumption throughout the study duration [Figure 2]. This indicates that Muno-IgY® did not cause drastic changes in the overall microbial activity. Consistently, lactate levels remained low and similar to the control throughout the experimental period [Figure 3A].

#### SCFA production

Compared to the control, Muno-IgY® treatment led to a significant increase in acetate [Figure 3B] and propionate [Figure 3C] production from day 3 onward (*P* < 0.05), while butyrate [Figure 3D] levels showed a decreasing trend over time. On day 8, acetate and propionate production significantly increased by 66% and 76%, respectively. In contrast, on day 8, butyrate production decreased significantly by 41% compared to the control.

#### Ammonium concentration

**Figure 4:**
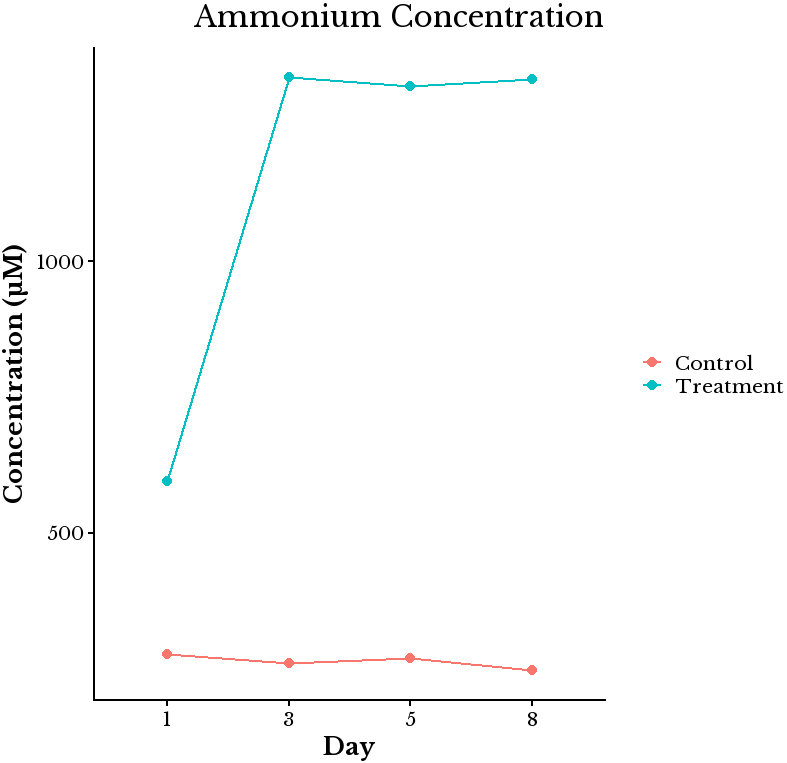
Effect of Muno-IgY® treatment on ammonium concentration in SHIME® reactors. Ammonium levels were measured on days 1, 3, 5, and 8 of treatment. IgY supplementation significantly increased ammonium concentration by day 8 compared with control reactors, suggesting enhanced proteolytic fermentation or microbial degradation of IgY (*n* =1). IgY: Immunoglobulin Y.

A significant increase in ammonium levels (443%) was observed in the Muno-IgY®-treated reactors compared to the control on day 8 (*P* < 0.01), suggesting increased proteolytic fermentation or microbial degradation of IgY [Figure 4].

#### Microbial community composition

On day 8, the Muno-IgY® treatment significantly increased the bacterial biomass (9.5 log_10 _CFU/mL) compared to the control (9.2 log_10_ CFU/mL) (*P* < 0.05). Alpha diversity metrics (Shannon and Simpson indices) were calculated for the Lumen and Mucus regions to evaluate microbial richness and evenness between the Control and Muno-IgY® treatments. In the Lumen, the Shannon index was slightly higher in the Muno-IgY® group (2.79 ± 0.01) compared to the control (2.71 ± 0.004), indicating a marginal increase in microbial diversity with Muno-IgY® treatment. Similarly, the Simpson index was higher in Muno-IgY® (0.853 ± 0.001) relative to the control (0.781 ± 0.0002). However, the differences between groups were not statistically significant (p = 0.1). In the Mucus, Shannon diversity was higher in the Muno-IgY® group (3.97 ± 0.02) compared to the control (3.87 ± 0.016), and the Simpson index was also elevated (0.953 ± 0.0003 *vs.* 0.933 ± 0.001), suggesting a trend toward increased microbial diversity in the mucus layer following Muno-IgY® treatment. These differences were not statistically significant (*P* = 0.1).

**Figure 5:**
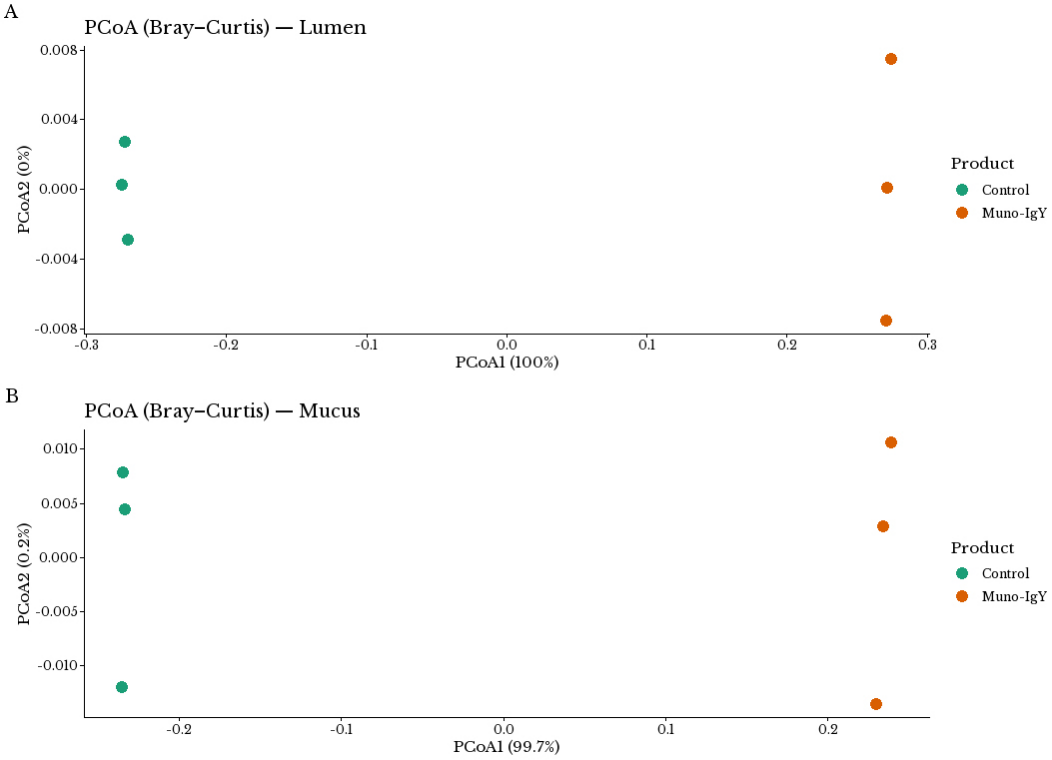
Effect of Muno-IgY® on the Beta-diversity of the treatment groups. Principal coordinates analysis (PCoA) was performed on Bray-Curtis dissimilarity matrices to evaluate the effect of Muno-IgY® on the β-diversity. Statistical differences in overall community composition were tested using PERMANOVA. No significant separation was observed between Control and Muno-IgY® groups in either the (A) lumen (*P* = 0.1) or (B) mucus (*P* = 0.1) compartments. IgY: Immunoglobulin Y. PERMANOVA: Permutational multivariate analysis of variance.

Beta diversity was assessed using Bray-Curtis distances followed by Permutational multivariate analysis of variance (PERMANOVA) to examine differences in overall community composition between treatments in the lumen [Figure 5A] and mucosa [Figure 5B]. In the Lumen, PERMANOVA indicated no statistically significant difference between control and Muno-IgY® groups (*R*² = 0.9995, *P* = 0.1). In the Mucus, PERMANOVA also showed no significant separation between groups (*R*^2^ = 0.9972, *P* = 0.1). These results suggest that while Muno-IgY® treatment may slightly increase microbial diversity, it does not significantly alter overall microbial community structure in either the lumen or mucus layers under the tested conditions.

**Figure 6:**
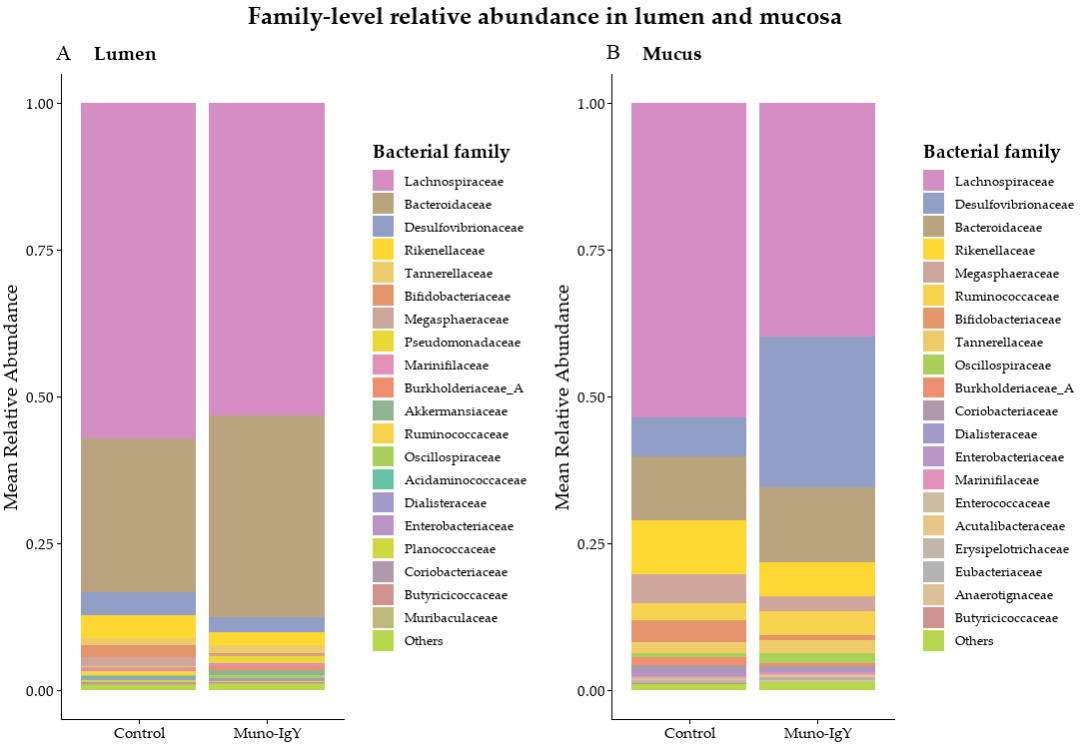
Family-level relative abundance in lumen and mucosal compartments. Relative abundance was calculated from normalized sequencing reads and expressed as the mean proportion per treatment group. (A) Luminal microbiota composition showing mean relative abundance of the top 20 bacterial families across control and Muno-IgY® treatments, with remaining taxa grouped as “Others”; (B) Mucosal microbiota composition showing mean relative abundance of the top 20 bacterial families across control and Muno-IgY® treatments, with remaining taxa grouped as “Others”. No taxa reached statistical significance following false discovery rate (FDR) correction, therefore, differences are presented descriptively. IgY: Immunoglobulin Y.

Microbial community profiling revealed distinct IgY-induced shifts in both the luminal and mucosal environments. The top twenty bacterial species, in terms of relative abundance, in the luminal and mucosal compartments of the control and Muno-IgY treatment groups are shown in Figure 6A and B, respectively. Although no taxa reached statistical significance after false discovery rate (FDR) correction, the observed fold changes (|log_2_FC| ≥ 2) are considered biologically meaningful because they represent large relative shifts in specific taxa that are functionally important.

**Figure 7:**
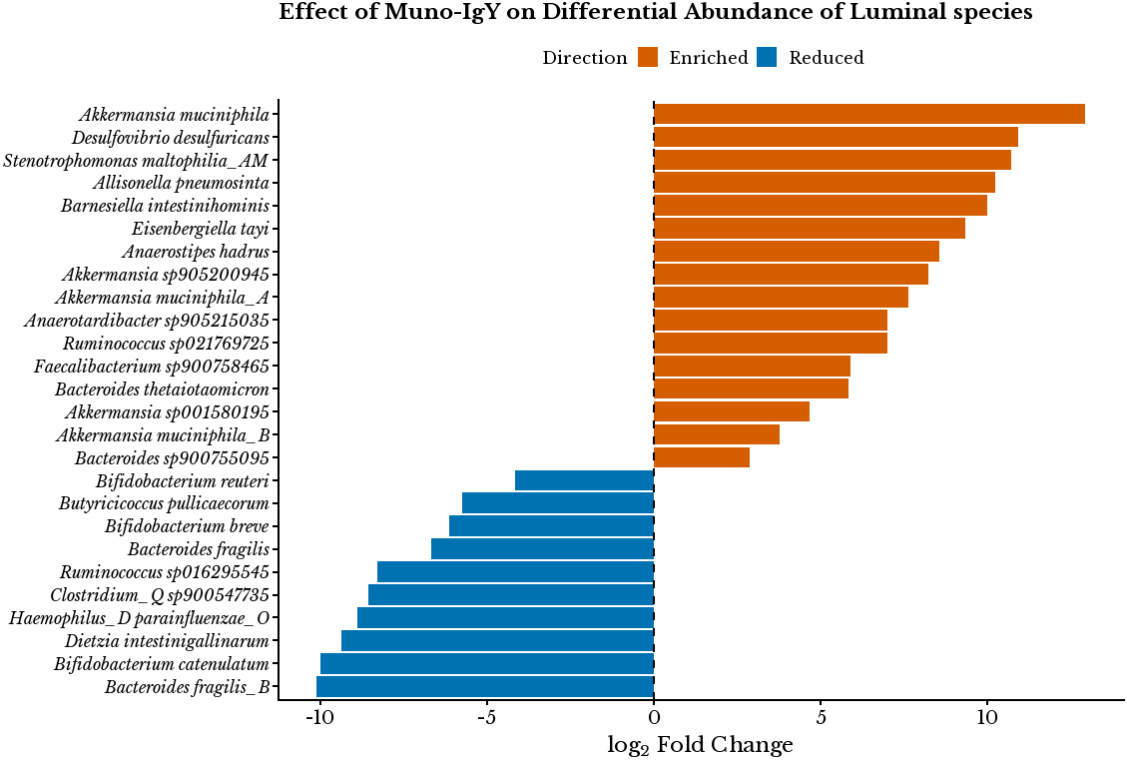
Differential abundance of selected microbial species in the luminal compartment. log_2_FC values were calculated as the ratio of mean relative abundance in the Muno-IgY® group relative to the control. Positive log_2_FC values indicate enrichment in the Muno-IgY® group, whereas negative values indicate depletion relative to Control. Findings are interpreted as descriptive shifts rather than inferential outcomes. IgY: Immunoglobulin Y; log_2_FC: log_2_ fold change.

In the luminal compartment, the Muno-IgY® treatment displayed a marked enrichment of several beneficial commensals and mucin-degrading species [Figure 7]. Notably, *Akkermansia muciniphila* (log_2_FC = 12.93), *A. muciniphila_A* (7.62), *A. muciniphila_B* (3.77), and *Akkermansia sp. 905200945* (8.23) and Barnesiella intestinihominis (10.00) were markedly increased, which are known to support mucosal barrier integrity and gut homeostasis. Similarly, SCFA producers, including *Anaerostipes hadrus* (8.54), *Anaerotardibacter sp. 905215035* (7.00), *Allisonella pneumosinta* (10.2), and *Ruminococcus sp. 021769725* (6.98) were also enriched, suggesting enhanced saccharolytic fermentation and intestinal metabolic activity. A remarkable increase was also observed in sulphate-reducing and electron-accepting species such as *Desulfovibrio desulfuricans* (10.90) and *Stenotrophomonas maltophilia_AM* (10.71). Conversely, several taxa typically associated with commensal gut functions or opportunistic pathobionts were reduced. These included *Bacteroides fragilis* (-6.69), *B. fragilis_B* (-10.11), *Bifidobacterium breve* (-6.12), *B. reuteri* (-4.15), *B. catenulatum* (-9.99), *Ruminococcus sp. 016295545* (-8.30), and *Clostridium_Q sp. 900547735* (-8.55). Notably, *Bacteroides thetaiotaomicron* (log_2_FC = 5.83) and *Bacteroides sp. 900755095* (2.86) were markedly elevated, indicating potential enrichment of species capable of complex carbohydrate degradation and mucin utilization.

**Figure 8:**
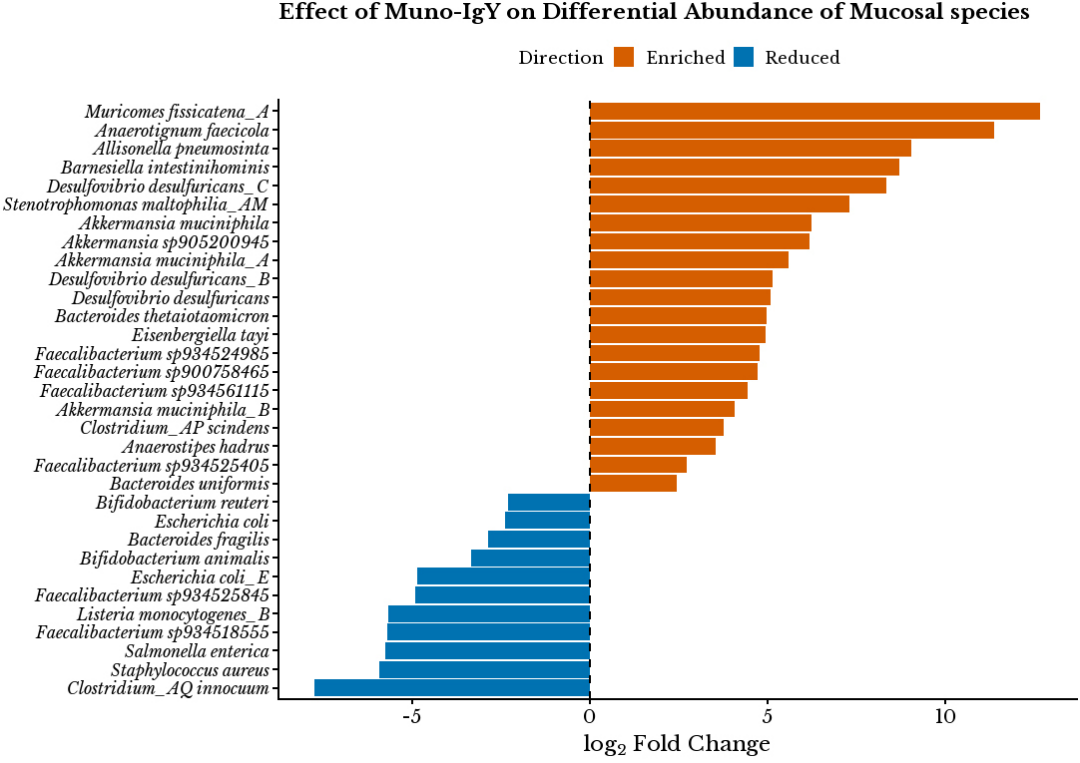
Differential abundance of selected microbial species in the mucosal compartment. log_2_FC values were calculated as the ratio of mean relative abundance in the Muno-IgY® group relative to the control. Positive log_2_FC values indicate enrichment in the Muno-IgY® group, whereas negative values indicate depletion relative to Control. Findings are interpreted as descriptive shifts rather than inferential outcomes. IgY: Immunoglobulin Y; log_2_FC: log_2_ fold change.

In the mucosal compartment, similar enrichment patterns were observed [Figure 8]. *Akkermansia* sp905200945 (6.20), *Akkermansia muciniphila* (6.24), *A. muciniphila*_A (5.59), and *A. muciniphila*_B (4.07) were enriched, along with *Barnesiella intestinihominis* (8.71), indicating reinforcement of mucosal barrier-associated taxa. SCFA producers such as *Anaerostipes hadrus* (3.55). Notably, *Anaerotignum faecicola* (11.40), *Eisenbergiella tayi* (4.96), and *Muricomes fissicatena*_A (12.70) were strongly enriched, reflecting augmented anaerobic fermentative activity at the epithelial interface. In contrast, several opportunistic or pathogenic taxa were suppressed, including *E. coli* (-2.39), *E. coli*_E (-4.85), *Clostridium_AQ innocuum* (-7.76), *Salmonella enterica* (-5.75), *Staphylococcus aureus* (-5.94), and *Listeria monocytogenes*_B (-5.68).

### Caco-2/PBMC co-culture with SHIME® effluent

**Figure 9:**
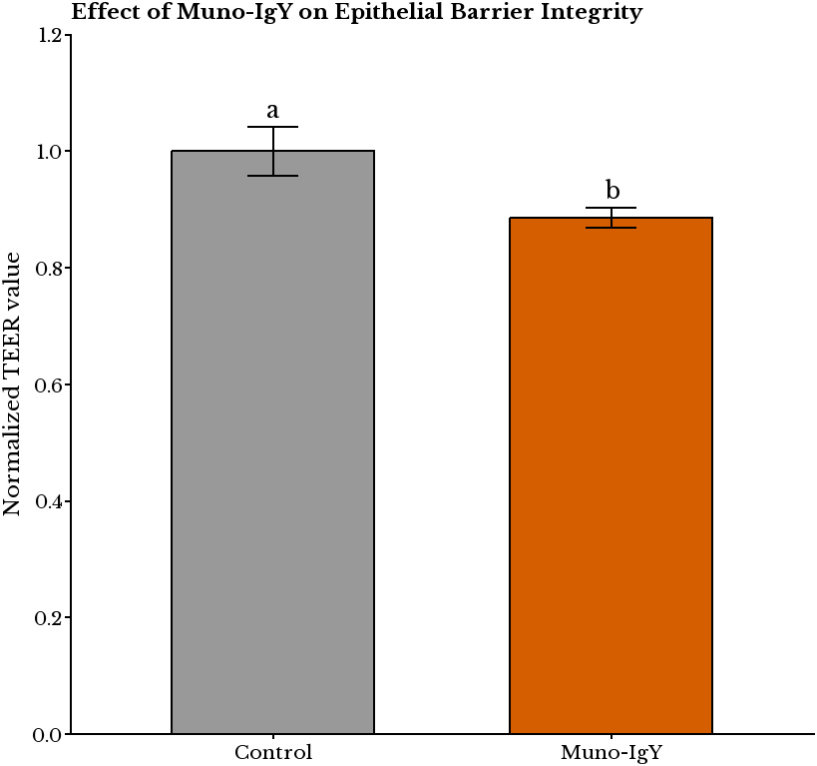
Effect of Muno-IgY® on the epithelial barrier integrity of Caco-2/PBMC cells. TEER was measured in Caco-2/PBMC co-cultures after exposure to SHIME® day 8 effluents. Statistical significance was assessed by a paired t-test (*P* < 0.05). Values are expressed as mean ± SD normalized to control (*n* = 9 replicates). Different letters above bars indicate statistically significant differences between treatments. TEER: Transepithelial electrical resistance; IgY: immunoglobulin Y; PBMC: peripheral blood mononuclear cell; SD: standard deviation.

To assess downstream effects of the SHIME®-modulated microbiota, day-8 reactor suspensions were applied to a Caco-2/PBMC co-culture. After 48 h of incubation, the TEER measurements showed that while the control SHIME® effluent preserved barrier integrity under inflammatory stimulation, the IgY-treated effluent resulted in a significant reduction in TEER values after 48 h, indicating compromised barrier function (0.886 ± 0.039 *vs.* 1.000 ± 0.000; *P* = 1.65×10^-5^) [Figure 9].

**Figure 10:**
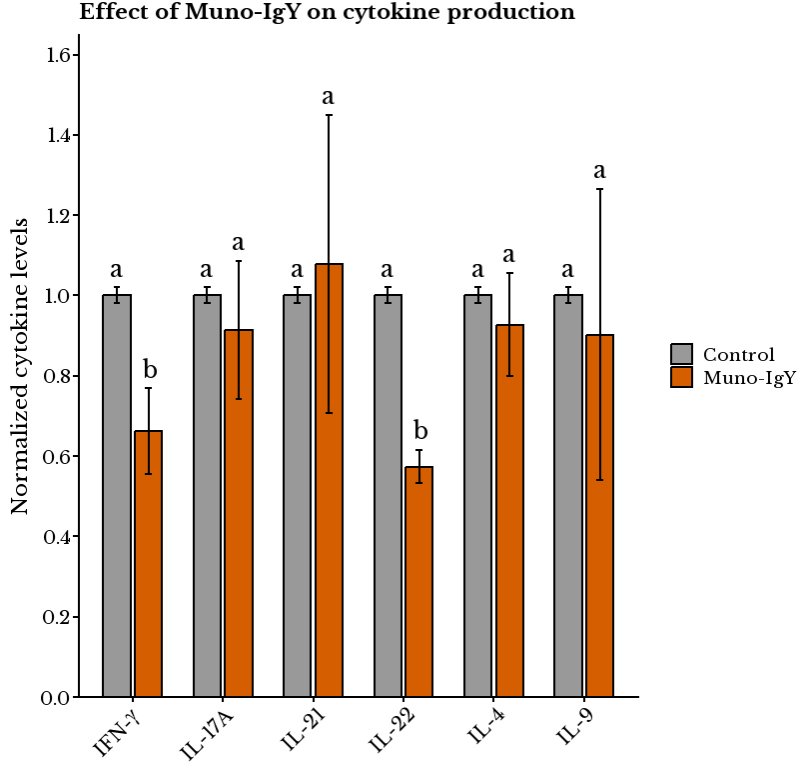
Effect of Muno-IgY® on cytokine production. Cytokine levels were measured in Caco-2/PBMC co-cultures exposed to SHIME® day8 effluents. Statistical significance was assessed by a paired *t*-test (*P* < 0.05). Values are expressed as mean ± SD normalized to control (*n* = 9 replicates). Different letters above bars indicate statistically significant differences between treatments. IFN-γ: Interferon-γ; IL-17A: Interleukin-17A; IL-21: Interleukin-21; IL-22: Interleukin-22; IL-4: Interleukin-4; IL-9: Interleukin-9; IgY: immunoglobulin Y; PBMC: peripheral blood mononuclear cell; SD: standard deviation.

Cytokine analysis of the basolateral supernatants revealed that Muno-IgY® treatment significantly reduced IFN-γ (0.662 ± 0.098 *vs.* 1.000 ± 0.063, *P* = 2.37×10^-5^) and IL-22 (0.574 ± 0.075 *vs.* 1.000 ± 0.043, *P* = 4.31×10^-7^) across all three PBMC donors compared to control [Figure 10]. Changes in IL-4 (0.928 ± 0.128, *P* = 0.20), IL-9 (0.905 ± 0.378, *P* = 0.51), IL-17A (0.913 ± 0.165, *P* = 0.18), and IL-21 (1.078 ± 0.340, *P* = 0.53) were not statistically significant. Additionally, reductions in IL-9 and IL-4 were observed in each donor.

## DISCUSSION

The intestinal microbiota plays a central role in host health, but its inaccessibility *in vivo* makes *in vitro* models essential for studying microbial interactions and therapeutic interventions. Dynamic fermentation models, such as the SHIME®, provide valuable insights into how candidate interventions affect gut microbial ecology^[11,15]^, while epithelial and immune co-culture systems complement these models by simulating downstream host responses. In contrast to previous studies that have primarily examined pathogen- or toxin-specific IgY in isolated infection models, the present work expands the scope of IgY evaluation to include microbiome- and host-associated readouts within a complex simulated gut ecosystem. Across multiple experimental systems, Muno-IgY® was observed to inhibit bacterial growth and adhesion, alter the relative abundance of specific taxa without significantly changing overall microbial community structure, and modulate host immune responses. Together, these findings are consistent with the hypothesis that multi-pathogen-specific IgY formulations may exert effects beyond direct pathogen neutralization, potentially involving indirect regulation of the gut microbial ecosystem and host immune responses. However, causal relationships and underlying molecular mechanisms were not directly interrogated and therefore cannot be concluded from the present data.

In the Caco-2 adhesion/invasion assay, Muno-IgY® treatment was associated with reduced interaction between the AIEC and enterocytes. This finding aligns with previous studies indicating that IgY can reduce the adhesion of enteric pathogens, such as *Salmonella* on Caco-2 cells^[20]^ and *Helicobacter pylori* on GES-1 cells^[21]^, in a concentration-dependent manner. These observations are consistent with supplementary growth inhibition assays, in which Muno-IgY® delayed *E. coli* proliferation in both broth and agar-based systems, particularly when administered during early growth phases [Supplementary Figures 1 and 2]. However, the exact mechanism of action by which IgY mediates its protective effects is not fully known. One possible mechanism is that IgY binding to bacterial surface antigens may sterically hinder epithelial attachment or impair bacterial motility. In the case of Muno-IgY®, unpublished binding data indicate reactivity toward *E. coli* flagellin, which could contribute to reduced adhesion and invasion. In addition, interference with flagellin-mediated signaling could attenuate host inflammatory responses, further limiting pathogen-induced damage^[22,23]^. The absence of additional benefit at 6 mg/mL suggests saturation of antigenic targets at 3 mg/mL, marking this as the effective dose for further applications.

While the Caco-2 assays provided insight into the effect of Muno-IgY® on direct host-pathogen interactions, the SHIME® model enabled us to study how repeated IgY exposure affects the gut microbial community. The SHIME® experiment was conducted using fecal material from a single healthy donor selected for high Enterobacteriaceae abundance, enabling sensitive detection of IgY-associated shifts in a targeted microbial context. While this approach allowed controlled and tractable evaluation of IgY-driven microbial shifts, it also limits the generalizability of the microbiome findings. SHIME® outcomes are known to vary substantially based on donor microbiome composition, diet, and metabolic profile; therefore, the observed changes in taxonomic abundance and SCFA production should be interpreted as donor-specific and exploratory. Additional studies using multiple donors with diverse baseline microbiota will be essential to confirm the consistency and robustness of the microbiome-modulatory effects observed in this study.

Within this framework, Muno-IgY® supplementation was associated with a modest increase in alpha diversity in both lumen and mucus. The lack of significant differences in beta diversity indicates that while diversity indices are modestly increased, the overall microbial community structure remains largely stable. This stability is important in maintaining gut homeostasis and suggests that Muno-IgY® does not disrupt the existing microbial ecosystem but may instead fine-tune the relative abundance of specific taxa^[24]^. Muno-IgY® supplementation was also associated with shifts in taxonomic composition, including enrichment of several taxa commonly associated with gut health. These included *Akkermansia muciniphila *and* Barnesiella intestinihominis* in the luminal and mucosal compartments, *Anaerostipes hadrus *and* Bacteroides thetaiotaomicron* in the luminal compartment, and *Muricomes fissicatena* in the mucosal compartment. *Akkermansia muciniphila* is a mucin-degrader associated with improved glucose homeostasis, intestinal integrity, reduced metabolic endotoxemia, and inflammation^[25]^.* B. intestinihominis* is known to utilize complex-type *N*-glycan as a carbon source in the human gut, while *B. thetaiotaomicron* is a major bacterial species of the human intestine that has multiple polysaccharide utilization loci and is capable of metabolizing mucins^[26]^. Although these associations are well documented, the present data do not establish direct functional consequences of these compositional changes.

In addition to enriching beneficial taxa, Muno-IgY® supplementation was also associated with reduced abundance of taxa often described as opportunistic or pro-inflammatory, such as *E. coli *and *Salmonella enterica*, which are common causes of inflammation and diarrhea in humans^[27]^. These observations suggest that Muno-IgY® may selectively limit expansion of pathobiont populations while sparing or promoting commensal taxa.

Recent studies investigating IgY have primarily focused on its ability to neutralize individual pathogens or toxins in simplified systems. For example, pathogen-specific IgY has been shown to reduce the adhesion of *Salmonella*^[28,29]^ and *Helicobacter pylori*^[21]^ to epithelial cells and to attenuate inflammatory responses in animal infection models. In contrast, the present study extends these findings by examining the effects of Muno-IgY® within a dynamic microbial community under simulated colonic conditions and linking microbial shifts to downstream host immune responses. Notably, enrichment of taxa such as *Akkermansia muciniphila*^[30]^ and *Barnesiella intestinihominis*^[31]^ observed here aligns with prior reports linking these organisms to enhanced barrier function and colonization resistance. Discrepancies across studies may reflect differences in IgY specificity, dosing, exposure duration, and baseline microbiota composition. These considerations underscore the context-dependent nature of IgY-microbiota interactions.

Finally, in the Caco-2/PBMC co-culture model, SHIME® effluents enabled an exploratory assessment into microbiota-host crosstalk. Under inflammatory conditions, effluents from IgY-treated reactors were associated with reduced TEER values, suggesting compromised barrier integrity, while simultaneously inducing an anti-inflammatory cytokine profile. The decrease in TEER value might be correlated to the decrease in the abundance of butyrate-producing taxa and the corresponding decline in butyrate concentration in the effluent. Butyrate is a key energy source for colonocytes and supports tight junction integrity, and hence decreases in butyrate availability may transiently weaken epithelial barrier function and lower TEER values, even while inflammation is dampened^[32]^. Since the SHIME study was exploratory and designed for short-term treatment, the fluctuation of the metabolites may be temporary. Further study employing multiple donors with repeated measurements over a longer term could address the observation.

Consistent with this interpretation, pro-inflammatory cytokines IFN-γ and IL-22 were significantly reduced across all PBMC donors, while decreases in IL-9 and IL-4 were observed in a subset of donors. These findings are consistent with previous murine *in vivo* studies, where oral administration of IgY during *Salmonella* and Enterotoxigenic *E. coli* infection similarly attenuated inflammation by downregulating pro-inflammatory cytokines and enhancing anti-inflammatory cytokine expression^[33,34]^.

Taken together, this multi-tiered study provides descriptive evidence that Muno-IgY® supplementation is associated with reduced *E. coli* epithelial adhesion/invasion, modulation of gut microbiome and host immune responses. These findings are consistent with a model in which IgY may influence host-microbe interactions at multiple levels, including direct effects on bacterial behavior and indirect effects mediated through microbial community shifts. However, the data do not establish causality or define underlying molecular mechanisms by which IgY mediate the observed effects.

However, this study has several limitations that should be considered when interpreting the findings. All experiments were conducted using *in vitro* models, which, while highly controlled and mechanistically informative, cannot fully capture the complexity of the human gastrointestinal environment, including host physiology, immune signaling, and dietary variability. The SHIME® experiments were performed using microbiota derived from a single donor, which limits the generalizability of the results across diverse microbiome profiles. Fermentation activity was primarily assessed using pH-related parameters and metabolite profiling, rather than direct measurement of gaseous fermentation products such as hydrogen, methane, or hydrogen sulfide, which could provide additional insight into microbial metabolic activity. In addition, the study focused on short-term Muno-IgY® exposure and did not assess long-term microbiome adaptation. Finally, while the findings provide testable hypotheses for IgY-mediated modulation of microbial and host responses, *in vivo* validation will be required to confirm physiological relevance and clinical applicability.

### Conclusion

This study demonstrated that Muno-IgY® acts across multiple levels of the gut ecosystem. It reduces AIEC adhesion and invasion, supporting its role in targeting pathobiont behavior at the epithelial interface. At the microbial level, it does not disrupt overall community structure but is associated with selective shifts in taxa and changes in fermentation profiles, including increased acetate and propionate alongside reduced butyrate. These changes suggest a modulatory, rather than disruptive, effect on the microbiome. Importantly, these microbial shifts translate into measurable host responses. While IgY-treated effluents reduced pro-inflammatory cytokine production, they were also associated with decreased epithelial barrier integrity under inflammatory conditions, highlighting a trade-off that warrants further investigation. Taken together, Muno-IgY® shows potential as a microbiome-aware passive immunotherapy that can influence pathogen behavior, microbial composition, and immune signaling. However, these findings remain exploratory and context-dependent. Future *in vivo* studies and multi-donor investigations are needed to clarify mechanisms, confirm reproducibility, and determine clinical relevance.

## DECLARATIONS

### Availability of data and materials

The raw data supporting the findings of this study are available within this Article and its Supplementary Materials. Further data are available from the corresponding authors upon request.

### AI and AI-assisted tools statement

During the preparation of this manuscript, the AI tool ChatGPT (OpenAI, GPT-5.3 family model, accessed on 27 November 2025) was used for language editing and generating the graphical abstract. The authors reviewed and edited all AI-generated content to ensure accuracy and compliance with journal standards and accept full responsibility for the final content of the manuscript.

### Copyright

© The Author(s) 2026.
