# Supplementary material for: Immunoglobulin Y protects intestinal epithelium and modulates gut microbiota
Source: Microbiome Res Rep. 2026 Apr 21;5(2):8. doi: 10.20517/mrr.2025.104 (PMC13246490; doi:10.20517/mrr.2025.104)
Supplement: Supplementary file 1 [file mrr-5-2-8-SupplementaryMaterials.pdf]

Immunoglobulin Y protects intestinal epithelium and modulates gut microbiota

Shahna Fathima[1], Tina Sarkar[1,3], Navneet Sharma[1], Paul E Kilgore[2], Huan H Nguyen[1]

[1]IGY Immune Technologies & Life Sciences, Airdrie T4A 2G8, Canada.
[2]Eugene Applebaum College of Pharmacy and Health Sciences, Wayne State University, Detroit, MI 48201, USA.
[3]Department of Dermatology, University Medical Center of the Johannes Gutenberg University, Mainz 55131, Germany.

Correspondence to: Dr. Shahna Fathima, IGY Immune Technologies & Life Sciences, Airdrie T4A 2G8, Canada. E-mail: sfathima@igylifesciences.com

ORCID: Shahna Fathima (0000-0003-1263-0870)

Supplementary Data

Bacterial growth inhibition assay

Since Muno-IgY® contains IgY directed against *Escherichia coli* along with other pathogens, *E. coli* strains were used to evaluate the effect of Muno-IgY® on bacterial growth, adherence, and invasion. A single colony of *E. coli* OP50 was inoculated into Luria-Bertani broth (VWR® Canada, 89500-596) and incubated overnight at 37 °C under aerobic conditions. The following day, the culture was diluted in fresh LB broth to an OD_{600} of 0.15 and distributed into five sterile 15 mL culture tubes, each containing 9 mL of the diluted culture. Muno-IgY® was added at defined timepoints, and concentrations as follows: Tube 1- 10 mg Muno-IgY® added during lag, early-log, and mid-log phases, Tube 2- 15 mg Muno-IgY® added during lag and early-log phases, Tube 3- 30 mg Muno-IgY® added during lag phase only, Tube 4- 15 mg Muno-IgY® added during lag and mid-log phases, Tube 5- *E. coli* OP50 without Muno-IgY® (positive control with 9 mL diluted culture and 1 mL LB broth), Tube 6- Muno-IgY® (30 mg) in LB broth without *E. coli*

(negative control), and Tube 7- LB broth only (blank). IgY was added at defined bacterial growth phases to assess whether antibody-mediated inhibition is most effective during early colonization versus established bacterial proliferation, which is relevant to prophylactic versus therapeutic use scenarios. Samples were collected hourly from each tube to measure OD_{600} and for plating during the first three hours of incubation. Plating was done on LB Agar prepared with LB broth and 1.5% agar. The first five hours of growth were monitored to capture early IgY-mediated effects on bacterial proliferation before nutrient depletion or stationary phase dynamics could confound interpretation.

The growth-inhibitory effects of Muno-IgY® under different dosing regimens against *E. coli* OP50 were evaluated in broth (OD_{600}) (Supplementary Figure 1) and on agar (CFU/mL) (Supplementary Figure 2) over the first five and three hours post-treatment, respectively. In the broth assay, OD_{600} continued to increase in all treatment groups, indicating that Muno-IgY® did not fully inhibit growth but slowed the bacterial proliferation compared with the untreated control (Tube 5). The most pronounced effect was observed in Tube 4 (15 mg Muno-IgY® added during lag and mid-log phases), which reached an OD_{600} of 0.47 at 5 hours, compared to 0.52 in Tube 1 (10 mg added at three growth phases) and 0.50 in Tube 3 (30 mg added during lag phase). Tube 6, which contained only Muno-IgY® without bacteria, exhibited detectable OD_{600}, reflecting background signal from the product itself rather than microbial growth.
In the agar assay, colony counts were similarly reduced in tubes treated with Muno-IgY®. At 3 hours, CFU counts in Tube 2 (15 mg added during lag and early-log phases) and Tube 3 (30 mg added during lag phase) were 69 and 170, respectively, compared to 400 in the control (Tube 5). Although Tubes 1 and 4 displayed CFU counts similar to the control at 3 hours (450 and 400, respectively), their earlier time points showed transient reductions in viability, suggesting that repeated or phase-specific IgY additions delay bacterial proliferation but do not fully suppress growth.

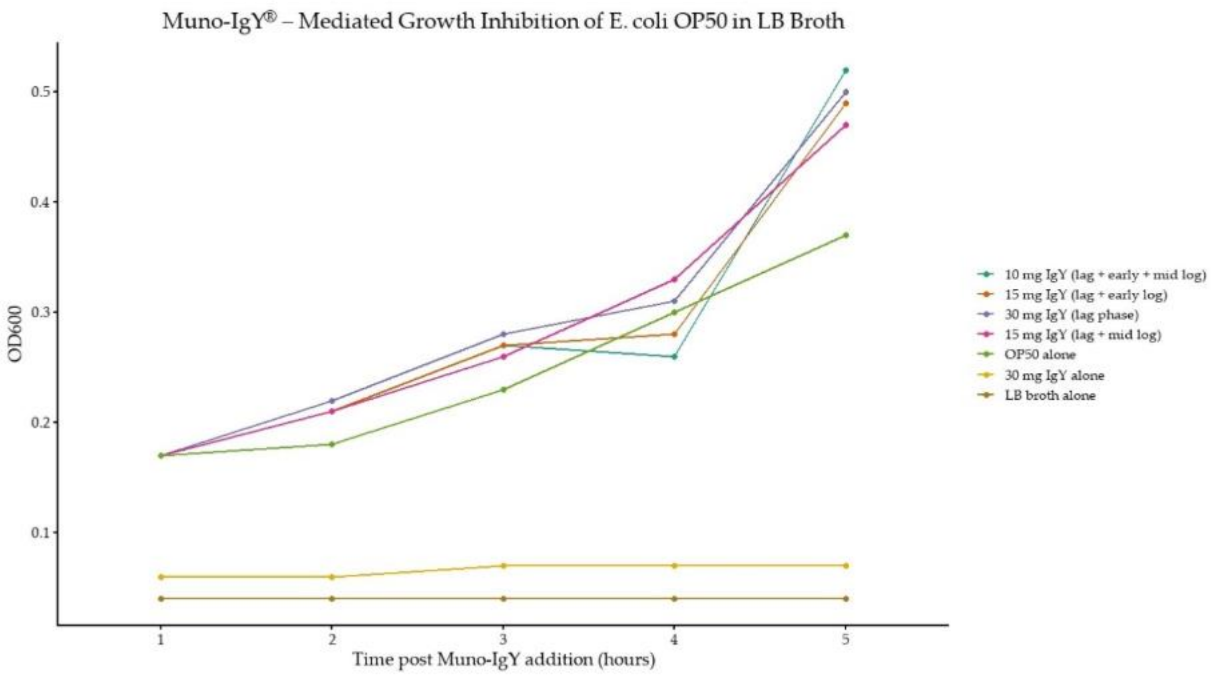

Supplementary Figure 1. Growth inhibition of *E. coli* OP50 in LB broth by Muno-IgY ® under different treatment conditions. Growth kinetics based on optical density (OD_{600}) measurements over five time points. Each line represents a different treatment tube. Data show mean absorbance (OD units) over time for each tube.

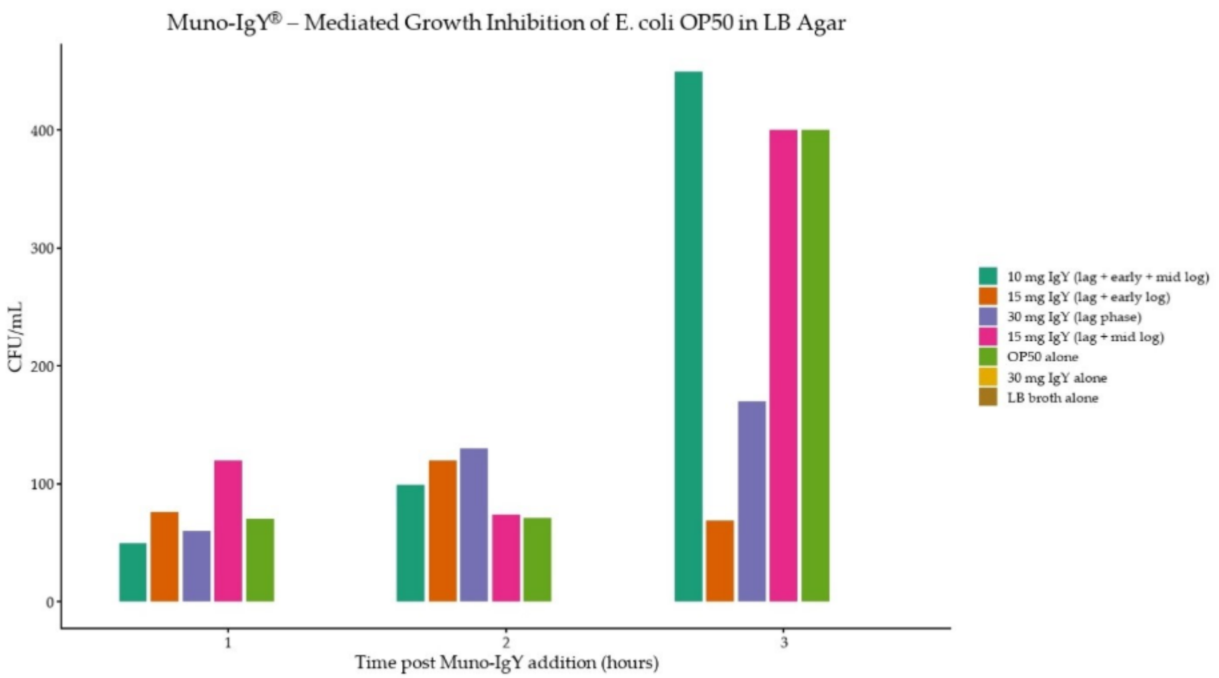

Supplementary Figure 2. Growth inhibition of *E. coli* OP50 in LB agar by Muno-IgY®

under different treatment conditions. Viable cell counts obtained from plating assays at three time points. Bars represent the number of colonies (CFU) for each treatment tube.
